# Establishing and maintaining fertility: the importance of cell cycle arrest

**DOI:** 10.1101/gad.348151.120

**Published:** 2021-05-01

**Authors:** Emily R. Frost, Güneş Taylor, Mark A. Baker, Robin Lovell-Badge, Jessie M. Sutherland

**Affiliations:** 1 Priority Research Centre for Reproductive Science, School of Biomedical Science and Pharmacy, School of Environmental and Life Sciences, University of Newcastle, Callaghan, New South Wales 2308, Australia;; 2 Hunter Medical Research Institute, New Lambton Heights, New South Wales 2305, Australia;; 3 Stem Cell Biology and Developmental Genetics Laboratory, The Francis Crick Institute, London NW1 1AT, United Kingdom

**Keywords:** Cip/Kip, gonad, granulosa, oocyte

## Abstract

Development of the ovary or testis is required to establish reproductive competence. Gonad development relies on key cell fate decisions that occur early in embryonic development and are actively maintained. During gonad development, both germ cells and somatic cells proliferate extensively, a process facilitated by cell cycle regulation. This review focuses on the Cip/Kip family of cyclin-dependent kinase inhibitors (CKIs) in mouse gonad development. We particularly highlight recent single-cell RNA sequencing studies that show the heterogeneity of cyclin-dependent kinase inhibitors. This diversity highlights new roles for cell cycle inhibitors in controlling and maintaining female fertility.

## Abstract (precis)

In this review, Frost et al. summarize the current knowledge on the Cip/Kip family of cyclin-dependent kinase inhibitors in mouse gonad development and highlight new roles for cell cycle inhibitors in controlling and maintaining female fertility.

Every organism relies on the cell division cycle for development. Maintaining the balance between cell differentiation and cell growth is essential in order for cells to access nutrients (Ruijtenberg and van den Heuvel 2016). Broadly, this balance shifts with age; during early development, rapid proliferation is required to create vital organs, but a reduction in proliferation is observed in many tissues with increasing age (Khosla et al. 2020). Driven by proteins such as cyclin-dependent kinases (CDKs) and cyclins, cells progress through the three phases of the cell cycle prior to mitosis and proliferation in M phase (Fig. 1). These phases, G_1_, S, G_2_, and M, are separated by tightly regulated checkpoints that must be passed for the cell to progress into the next phase. For example, at the G_1_ checkpoint, CDK proteins bind to cyclin D, which allows for the release of transcription factors to push the cell through to S phase (Sherr and Roberts 1999; Kanatsu-Shinohara et al. 2010; Granados-Aparici et al. 2019). For cells to exit the cell cycle and stop proliferating, CDKs need to be restricted. At the G_1_/S checkpoint, cyclin-dependent kinase inhibitors (CKIs) halt proliferation by binding to CDKs and initiating cell cycle arrest.

**Figure 1.**
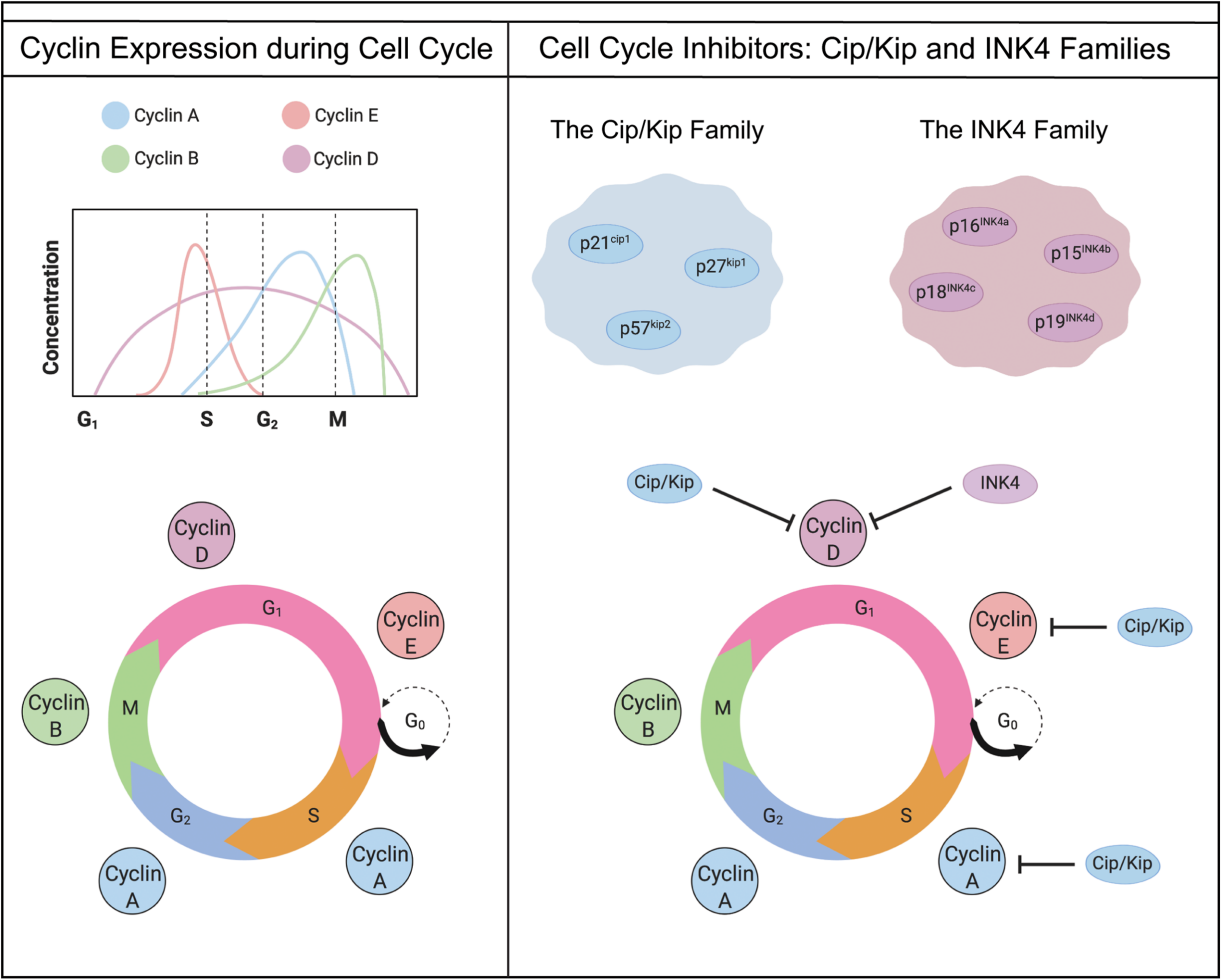
Phases of the cell cycle and the role of cell cycle inhibitors. The four phases of the cell cycle are G^1^, S, G_2_, and M phase. Cells must pass through the G_1_, S, and G_2_ phases to enter mitosis. At the G_1_ stage, cells can exit the cell cycle by entering the G_0_ phase. Cyclins are required to progress through the four cell cycle stages. Cyclins A, B, D, and E vary in expression level depending on the cell cycle stage. Cell cycle inhibitors are important regulators of cell cycle progression and proliferation. There are two main families of cyclin-dependent kinase inhibitors: the Cip/Kip family and the INK4 family. The Cip/Kip family consists of three proteins (p21^cip1^, p27^kip1^, and p57^kip2^), and the INK4 family consists of four proteins (p16^INK4a^, p15^INK4b^, p18^INK4c^, and p19^INK4d^). The Cip/Kip family is capable of inhibiting Cyclin A, Cyclin D, and Cyclin E, whereas the INK4 family can only inhibit Cyclin D. With the cyclin protein inhibited, the cell is unable to progress to the next stage of the cell cycle.

INK4 and Cip/Kip are two main families of cyclin-dependent kinase inhibitors. The INK4 family acts in a restricted manner as INK4 family members p16^INK4a^, p15^INK4b^, p18^INK4c^, and p19^INK4d^ prevent CDK4 and CDK6 binding to the D-type cyclins only (Sherr and Roberts 1999). Thus, after removing repressive INK4 proteins, cells immediately enter G_1_ phase by the action of Cyclin D. In contrast, the Cip/Kip family of cyclin-dependent kinase inhibitors bind all cyclins and CDKs and therefore have much more diverse functions (Nakayama and Nakayama 1998). The Cip/Kip family members—p21^Cip1^, encoded by the cyclin-dependent kinase inhibitor 1A (*Cdkn1a*) gene; p27^Kip1^, encoded by the cyclin dependent kinase inhibitor 1B (*Cdkn1b*) gene; and p57^Kip2^, encoded by the cyclin dependent kinase inhibitor 1C (*Cdkn1c*) gene—are traditionally associated with controlling cell divisions (Fig. 2; Li et al. 2012; Pippa et al. 2012; Jeannot et al. 2015; Orlando et al. 2015; Bicer et al. 2017). However, recent studies show that p21^Cip1^, p27^kip1^, and p57^kip2^ also regulate other cellular processes, including apoptosis, cell migration, cell fate decisions, and transcriptional regulation (for extensive reviews, see Sherr and Roberts 1999; Besson et al. 2008; Bachs et al. 2018). Specifically, in the mammalian gonads, recent technological advances are giving more insight into the importance of the cell cycle.

**Figure 2.**
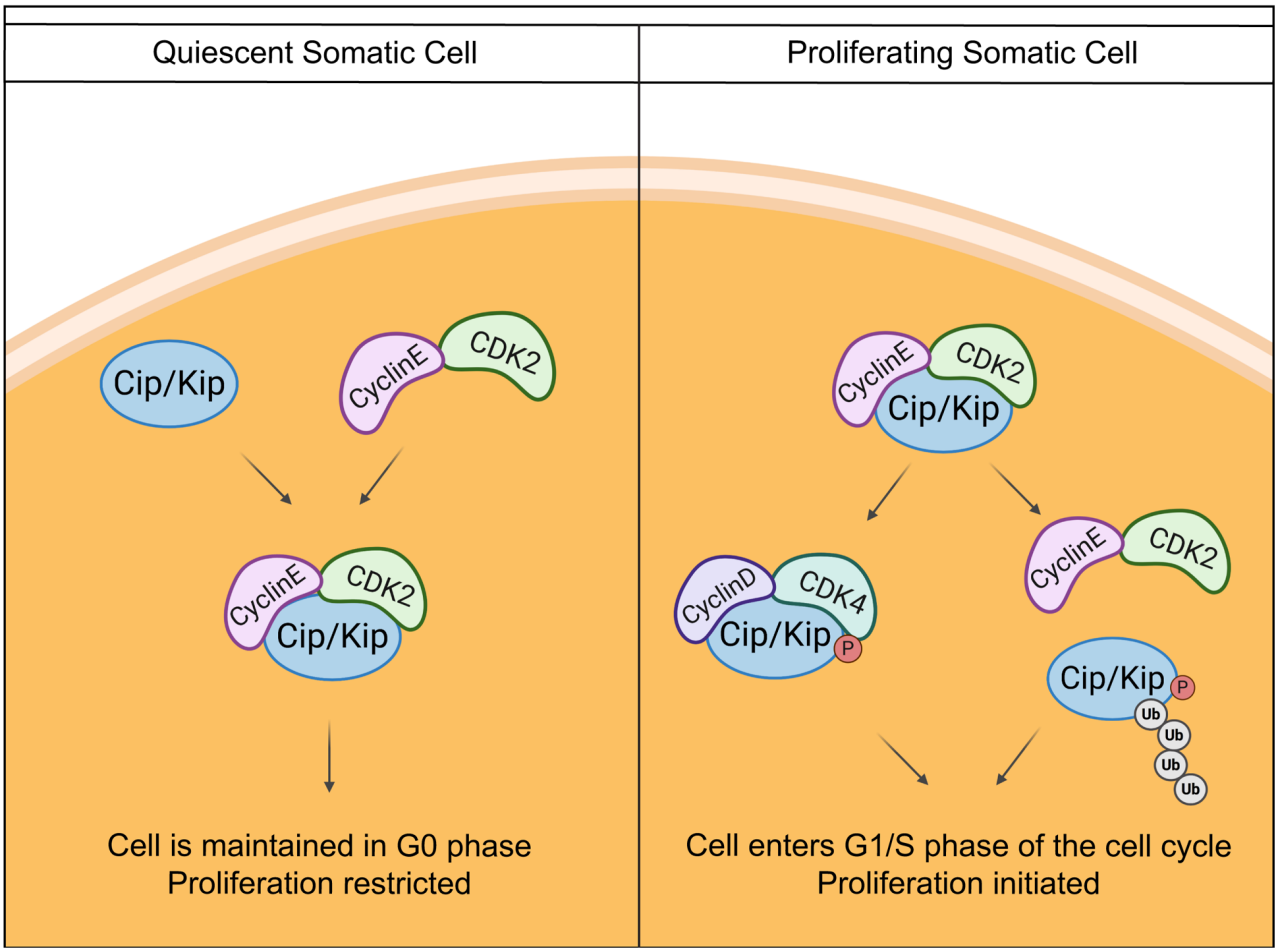
Canonical Cip/Kip protein action to regulate cell proliferation. The Cip/Kip family of proteins regulate cellular proliferation by inhibiting the action of CDKs. All three Cip/Kip proteins (p21^cip1^, p27^kip1^, and p57^kip2^) bind to CDK2 and Cyclin E to keep cells in the G_0_ phase of the cell cycle and maintain dormancy. When a cell receives signals to proliferate, Cip/Kip inhibition can be removed in multiple ways. Cip/Kip proteins become phosphorylated, which allows them to bind to Cyclin D and CDK4, which drives the cell to enter into G_1_ phase. Phosphorylation or acetylation of Cip/Kip proteins marks it for ubiquitination, and thus the protein is degraded by the proteasome. These post-translational modifications control Cip/Kip action to regulate cellular proliferation.

Mammalian gonads are unique organs as they contain not only somatic cells but also germ cells; hence, mitosis and meiosis occur in parallel within the same organ. This dynamic process must be tightly regulated to ensure successful transmission of genetic material to the next generation. Interestingly, changes in proliferation are one of the earliest differences between the ovaries and testes (Schmahl et al. 2000; Schmahl and Capel 2003). These differences persist into adulthood, when the testes are continually producing new sperm, but the ovaries are unable to generate new oocytes. Regulation of the cell cycle by CDKs and cyclins has been reviewed extensively in the testes (Wolgemuth and Roberts 2010; Lim and Kaldis 2013); however, recent studies reveal precise control of the cell cycle during ovarian development, highlighting the need for a new review of this field. Cip/Kip proteins are abundantly expressed in the early gonad, and importantly, deletion of the Cip/Kip family members in mice leads to unexpected gonad-specific fertility phenotypes in both sexes (Fero et al. 1996; Holsberger et al. 2005; Rajareddy et al. 2007; Lin et al. 2015). The primary focus of this review is on data obtained in mice as it is the model used in most recent reports pertaining to primary sex determination and early ovarian development. Details about the gene networks involved in ovarian somatic cell differentiation, mitotic and meiotic arrest in germ cells, and the changes in Cip/Kip protein expression during key ovarian transitions, e.g., from establishing the oocyte reserve to selective oocyte maturation, are presented (Fig. 3). Single-cell RNA sequencing permits the gathering of large amounts of information, both on gene expression and cell populations, in a single experiment. In particular, this technology has unveiled new cell types or subpopulations in nearly all tissues explored, expanding our understanding in its wake. By exploring these data sets with a cell cycle-centric view, it is evident there is still much to learn about cell cycle control in specifying cell fate in the developing gonad. By investigating the dynamic changes in Cip/Kip expression across mouse ovarian development, this review illustrates and emphasizes the importance of Cip/Kip proteins in establishing and maintaining the gonad, and ultimately ensuring fertility.

**Figure 3.**
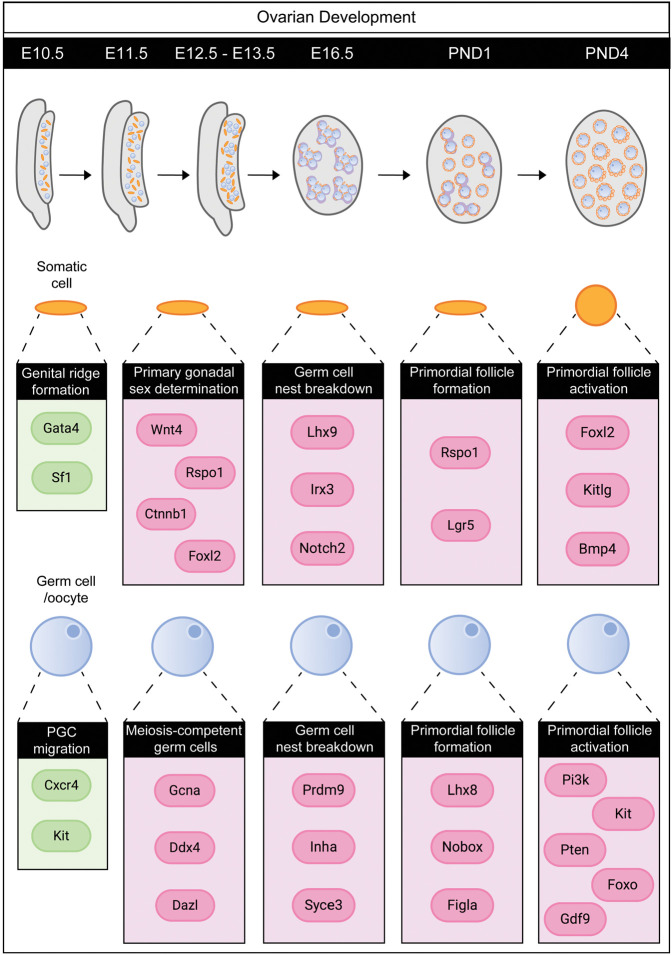
Key genes involved in the somatic cells and the germ cells during the development of the mouse ovary. The *top* panel shows diagrams of the mouse ovary during embryonic and early postnatal development, with the embryonic and postnatal day indicated in the black bar *above*. From E10.5, the gonad contains both the primordial germ cells (blue) and somatic supporting cell precursors (orange). A range of genes contribute to and participate in the gene regulatory networks controlling mouse ovarian development, which are shown in the *bottom* panel. Only a selection of genes shown to be critical for each developmental process are shown.

## Key genes drive primary sex determination in the somatic cells of XX and XY gonads

**Figure 4.**
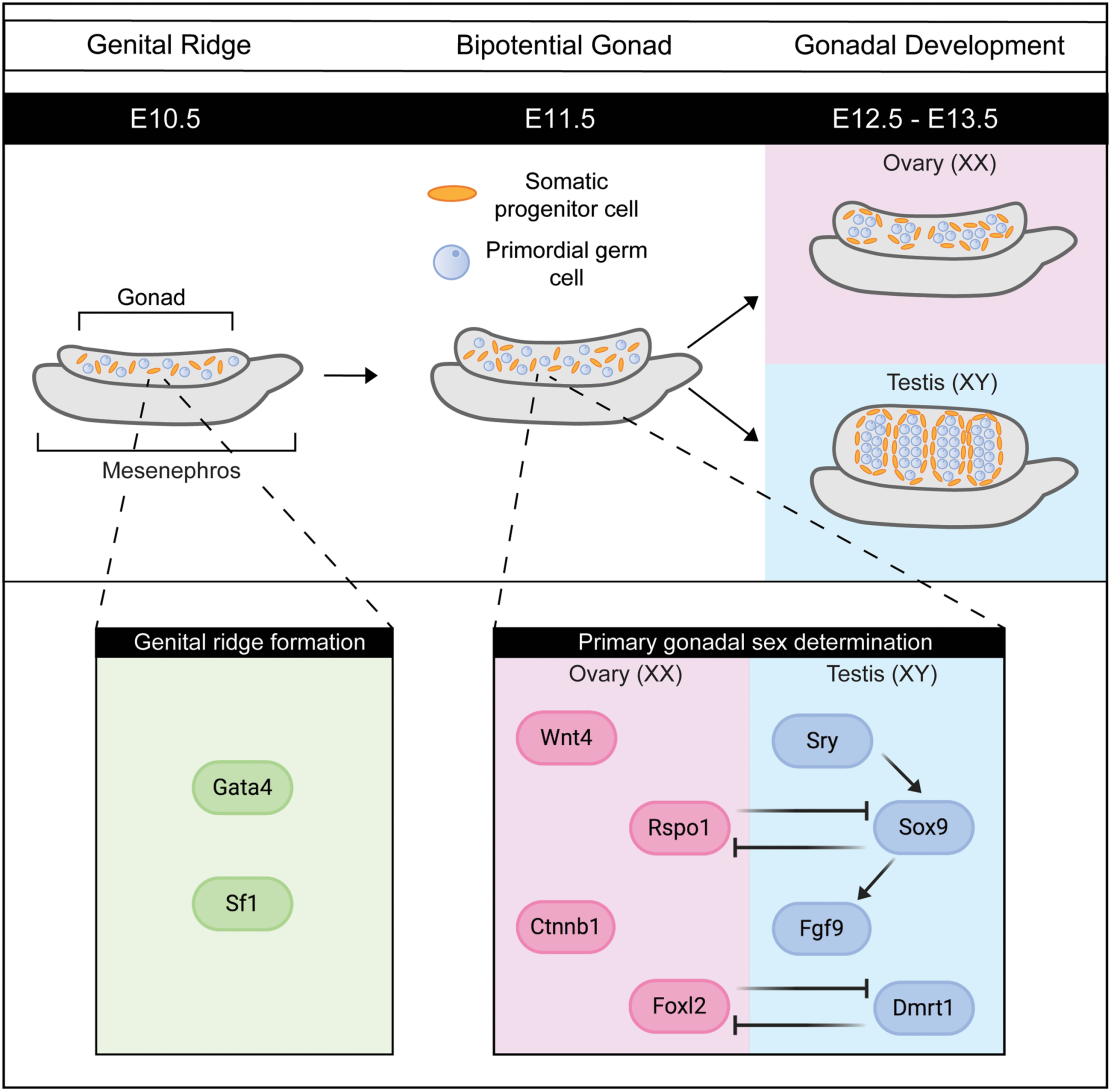
Factors controlling primary gonadal sex determination in mice. Around embryonic day 10.5 (E10.5) in mice, the genital ridge forms where the coelomic epithelium thickens. The transcription factors Gata4 and Sf1 are critical for the formation of the genital ridge and its expansion into the early gonad. By E11.5, the gonad contains both the primordial germ cells (blue) and somatic supporting cell precursors (orange), as well as precursors of steroidogenic cells and some connective tissue cells. At this time point, primary gonadal sex determination is initiated, driven by factors expressed in the supporting cell precursors. While testis determination is known to be initiated by one factor, Sry, and then orchestrated by Sox9, female gonadal sex determination is initiated by multiple factors. Wnt4, Rspo1, Ctnnb1, Runx1, and Foxl2, and perhaps others, all work to specify the ovarian fate (Ottolenghi et al. 2007; Gonen and Lovell-Badge 2019; Nicol et al. 2019). In the male, Sry stimulates expression of Sox9, which then up-regulates male-specific genes, including Fgf9 and Dmrt1 (Koopman et al. 1991; Kim et al. 2006). In both sexes, active repression of sex-specific factors maintains the gonadal identity (Uhlenhaut et al. 2009; Lindeman et al. 2015).

While mammalian sex is determined by XY male/XX female sex chromosomes, respectively, at fertilization, the formation of sex-specific gonads occurs later in embryonic development. This means that the mammalian gonads are a remarkable system to study cell fate decisions as, unlike other organs, the gonad develops as a bipotential organ with the ability to become either an ovary or a testis. In mice, gonads are specified from the coelomic epithelium around embryonic day (E) 9.5, where proliferating cells form a layer on the mesonephros (the embryonic kidney) (Hu et al. 2013). Less than 24 h later, at E10.3, this layer of cells on the mesonephros then thickens, forming the genital ridge (Fig. 4; Hu et al. 2013). Primordial germ cells concurrently migrate from the mesoderm into the genital ridge as it thickens (Richardson and Lehmann 2010). Genital ridge formation is dependent on the expression of GATA binding protein 4 (GATA4), which activates other early gonadal factors like LIM homeobox 9 (LHX9) and steroidogenic factor 1 (SF1) that sustain the continued growth of the genital ridge (Richardson and Lehmann 2010; Hu et al. 2013). Once the genital ridge thickens, this structure is known as a gonad, which contains both somatic cells and germ cells. The two critical somatic cell populations in XX and XY gonads, the supporting cells and steroidogenic cells, originate from a single progenitor cell lineage within the genital ridge (Stévant et al. 2018). Primary sex determination is the process by which cells of the supporting cell precursor lineage differentiate into Sertoli cells typical of the testis or pregranulosa cells of the ovary (Zhao et al. 2018; Stévant et al. 2019). These trigger other cell types, including the steroidogenic cells, germ cells, and connective tissue cells, to follow the testicular or ovarian pathway, thereby shaping the gonad and laying the foundations for future reproductive capacity. A failure of the gonads to develop as either XX ovaries or XY testes leads to infertility and disorders/differences of sex development (DSDs), often associated with infertility (Eozenou et al. 2020; Estermann and Smith 2020). The gene regulatory networks relevant to sex determination in mice are detailed in several previous reviews (Jakob and Lovell-Badge 2011; Capel 2017; Stévant and Nef 2019). Importantly for sex determination, the Y-encoded (male-specific) gene *Sry* is expressed in XY gonads and leads to the up-regulation of its critical target gene, SRY-related high-mobility group (HMG) box 9 (*Sox9*) (Koopman et al. 1991). High levels of SOX9 then stimulate the expression of additional genes required for testis development and for other aspects of the male phenotype, including fibroblast growth factor 9 (*Fgf9*), doublesex and Mab-3-related transcription factor 1(*Dmrt1*), and anti-Müllerian hormone (*Amh*) (Kim et al. 2006; Sekido and Lovell-Badge 2009; Jakob and Lovell-Badge 2011). In XX gonads, in the absence of *Sry*, there are multiple female-determining factors that work together, including Wnt family member 4 (*Wnt4*), β-catenin 1 (*Ctnnb1*), R-spondin precursor 1 (*Rspo-1*), and Forkhead box L2 (*Foxl2*) (Vainio et al. 1999; Ottolenghi et al. 2007; Chassot et al. 2014). Starting from the bipotential gonad, these key genes must work together to activate or repress genes to establish and then maintain gonad identity (Fig. 4). These early steps, between E11.0 and E11.5, are accompanied by hundreds of genes being up-regulated or down-regulated in XX and in XY gonads (Zhao et al. 2018; Stévant et al. 2019).

## Sex differences in cell cycle regulatory genes during gonadal sex determination

**Figure 5.**
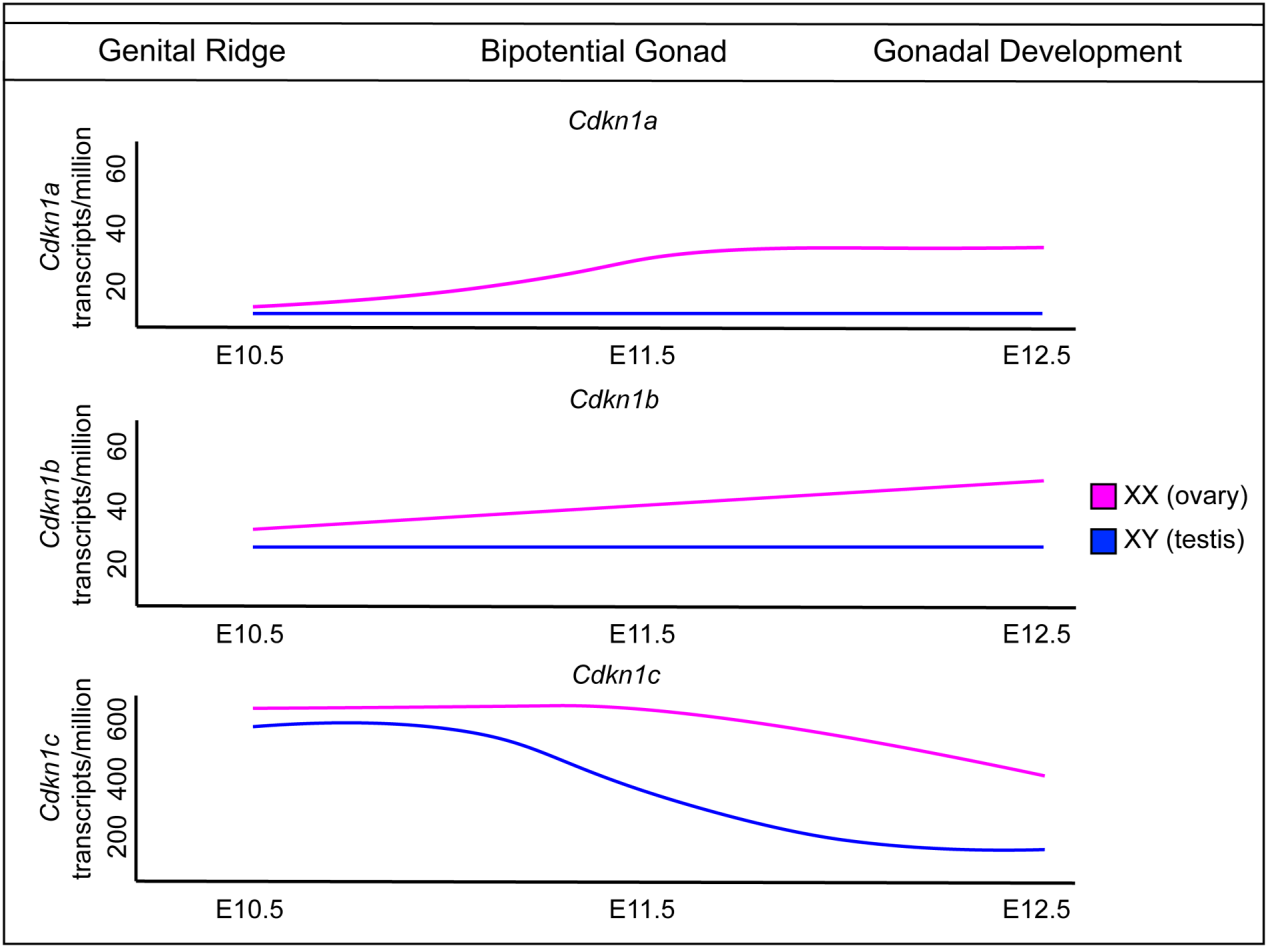
Expression of Cip/Kip transcripts across mouse gonadal sex determination. Around E10.5 when the genital ridge thickens, Cdkn1a, Cdkn1b, and Cdkn1c transcripts are all expressed in the somatic supporting cells (Zhao et al. 2018; Stévant et al. 2019). The genital ridge increases in size, forming the bipotential gonad. At E11.5, sex determination occurs, and the somatic supporting cells begin to differentiate into the XX or XY somatic cell lineages. In the testes, Cdkn1c is down-regulated in the somatic cells, and low levels of Cdkn1a and Cdkn1b are maintained, to allow the testes to increase in size to form the testis cords. In the ovary, Cdkn1a and Cdkn1b are up-regulated in the somatic supporting cells to maintain cells in dormancy. Germ cell expression of Cdkn1a, Cdkn1b, and Cdkn1c is currently unknown during sex determination. Gene expression graphs of Cdkn1a, Cdkn1b, and Cdkn1c transcripts during gonadal development are adapted from Zhao et al. (2018).

The advent of novel sequencing technologies such as single-cell RNA sequencing (scRNA-seq), chromatin immunoprecipitation sequencing (ChIP-seq), and assay for transposase-accessible chromatin sequencing (ATAC-seq) has accelerated our knowledge of the fundamental genes controlling many developmental processes (Stévant and Nef 2018, 2019; Estermann and Smith 2020; Tam and Ho 2020). These techniques have been applied, primarily in mice, to unravel the initiation of sex-determining pathways in mammalian gonads (Jameson et al. 2012; Garcia-Moreno et al. 2018; Stévant et al. 2018, 2019). Time-course analyses conducted before, during, and after sex determination, from E10.5 to E13.5, in both XX and XY gonads of mice (Stévant et al. 2018, 2019; Zhao et al. 2018) provide a wealth of temporal information. In this review, we integrate these publications to reveal novel facets of cell cycle regulation in the gonad and provide an in-depth characterization of Cip/Kip transcriptional expression (Fig. 5).

Interestingly, unlike the majority of genes expressed in the gonads, which are characteristically up-regulated at sex determination, transcriptomic studies show a baseline level of cyclin-dependent kinase inhibitor 1A (*Cdkn1a*), cyclin-dependent kinase inhibitor 1B (*Cdkn1b*), and cyclin-dependent kinase inhibitor 1C (*Cdkn1c*) transcript expression prior to sex determination and expression of *Sry* at E10.5 (Stévant et al. 2018, 2019; Zhao et al. 2018). These data suggest a baseline level of cyclin-dependent kinase inhibitors is required for supporting early gonad-specific factors during the growth of the genital ridges (Ikeda et al. 1994; Miyamoto et al. 2008; Hu et al. 2013). By E11.5, all three CKIs—*Cdkn1a*, *Cdkn1b*, and *Cdkn1c*—are expressed during sex determination; however, following supporting cell specification, their relative expression changes individually (Nef et al. 2005; Zhao et al. 2018). Each Cip/Kip CKI contains a CDK binding domain; however, large variability exists in the remainder of each protein's molecular structure (Nakayama and Nakayama 1998). *Cdkn1a* encodes the shortest protein (p21^cip1^), and p21^cip1^ interacts with proliferating cell nuclear antigen (PCNA), showing a direct inhibition of the cell cycle (Besson et al. 2008). p27^kip1^ and p57^kip1^ are longer proteins and have distinct domains, including the cyclin binding domain and CDK binding domain (Bachs et al. 2018). These two proteins also have linker domains and proline-rich domains with unknown functions, which may be implicated in the unique roles that these proteins are involved with during development (Nakayama and Nakayama 1998; Bachs et al. 2018). The expression of *Cdkn1a* and *Cdkn1b* transcripts increases fourfold and 1.4-fold, respectively, in the E11.5 developing ovary, so that these transcripts are elevated specifically in the XX gonad (Fig. 5; Zhao et al. 2018). *Cdkn1a* expression plateaus at E12.5; however, *Cdkn1b* transcript levels continue to rise as gestation continues (Zhao et al. 2018). The opposite trend is observed with *Cdkn1c* (p57^kip2^) transcript levels, where, by E12.5, *Cdkn1c* transcript is down-regulated in the XY gonad but maintained in the XX gonad (Stévant et al. 2018, 2019; Zhao et al. 2018). This is specifically restricted to the pregranulosa cell lineage, positive for the somatic cell marker steroidogenic factor 1 *Nr5a1* (Stévant et al. 2019). These data imply that expression of the Cip/Kip family, and thus cell cycle arrest, is functionally important for ovarian fate.

XX and XY gonads show substantial differences in cell proliferation immediately following sex determination (Schmahl et al. 2000), which are most likely attributed to transcriptional regulation. ChIP-seq experiments in recent years have identified novel interactions involving transcription factors that may control cell cycle arrest. Recent work by Nicol et al. (2018) performing ChIP-seq for the forkhead box L2 (FOXL2) protein in wild-type E14.5 mouse ovaries revealed that FOXL2 binds to an enhancer of *Cdkn1b*. Importantly, this FOXL2 peak overlaps with a RUNX1 ChIP-seq peak, illustrating co-operation, or at least redundancy, between ovarian factors to ensure the activation or repression of key target genes (Nicol et al. 2019). The *Cdkn1b* enhancer region is also bound by the protestis factors SOX9 and DMRT1 (Rahmoun et al. 2017; Nicol et al. 2018). These original findings suggest a dual approach, where p27^kip1^ expression is promoted by ovarian factors and repressed by testicular factors. Other experiments, where ectopic expression of FOXL2 is driven by the *Nr5a1* locus in the testis, result in the up-regulation of *Cdkn1b* transcript (Nicol et al. 2018). It is important to note that *Cdkn1b* is unlikely to be the only factor restricting proliferation in the ovary, and FOXL2 and SOX9 are involved in gene regulatory networks that must involve additional genes controlling and promoting proliferation. More interrogation of the CHIP-seq data sets is needed to map this network of cell cycle control in the gonad. Taken together, this indicates the male program represses *Cdkn1b* in the embryonic testis, but more importantly suggests that the key female factor FOXL2 is capable of promoting *Cdkn1b* expression. This information, when combined with the RNA sequencing data sets (Stévant et al. 2018, 2019), illustrates that expression of Cip/Kip family members in the supporting cell lineage contributes to the mechanisms of primary gonadal sex determination while they are in turn regulated by known players in this process.

Additional evidence supporting the regulation of Cip/Kip transcripts by ovarian-specific factors, including FOXL2, comes from genetic mouse models. When FOXL2 and WNT4 are deleted in mice, *Cdkn1b* transcripts and p27^kip1^ protein are down-regulated in the embryonic ovary (Garcia-Ortiz et al. 2009; Maatouk et al. 2013; Gustin et al. 2016). Conversely, when FOXL2 and RUNX1 (factors specific to pregranulosa cells) are genetically deleted, *Cdkn1a* expression increases (Nicol et al. 2018, 2019). This implies that cell cycle arrest is particularly important in the ovary following sex determination, and multiple factors act redundantly to ensure activation of cyclin-dependent kinase inhibitors. Whether the regulation of *Cdkn1a* and *Cdkn1b* by ovary-specific factors persists in the days following sex determination is currently unknown. Do ovarian factors continue to stimulate the transcription of *Cdkn1a* and *Cdkn1b*, or is their expression maintained through other downstream canonical cell cycle regulators? FOXL2, in particular, is known to become essential to maintain the ovary, because its postnatal deletion leads to gonadal sex reversal (Uhlenhaut et al. 2009). Uncovering when FOXL2 becomes the sole factor necessary for ovarian identity, without the help of other pathways, will lead to insights into what genes require consistent regulation throughout development.

## Sexual dimorphism is maintained in cell cycle regulatory genes following gonad differentiation

Once gonadal sex is specified around E11.5, the ovaries or testes must undergo many genetic and morphological changes to make them capable of maturing and supporting germ cells. Both the primordial germ cells, which have migrated into the gonad, and the somatic cells alter their cell cycle status in a sex-dependent manner. One of the earliest differences distinguishing ovary and testis development is the resumption of proliferation in the testes (Schmahl et al. 2000; Schmahl and Capel 2003). In mice, the change in cell cycle status presents as a clear morphological difference, where the testes are already double the size of the ovaries at E13.5 (Schmahl et al. 2000; Schmahl and Capel 2003; Nel-Themaat et al. 2009). This rapid proliferation is essential for forming embryonic testis cords, which eventually give rise to the seminiferous tubules of the adult testis. Pioneering work by Schmahl et al. (2000). showed that Sry activity (presumably via Sox9) is essential for promoting cell proliferation in the testis. Furthermore, when proliferation of the somatic cells is inhibited, just prior to sex determination (between E10.8 and E11.2), the testis is unable to correctly form testis cords and thus cannot support fertility in the adult male (Schmahl and Capel 2003). It is now widely accepted that Sertoli cells up-regulate the expression of key cell cycle genes to induce their proliferation (Rotgers et al. 2018; Stévant et al. 2018, 2019; Zhao et al. 2018).

Cip/Kip members were originally considered essential factors in the testis after sex determination, given that gonocytes, the germ cells at this stage of development, are held in mitotic arrest. However, there is now evidence that, at least in mice, Cip/Kip protein expression is variable- and strain-dependent. In the CD1 strain, p21^cip1^, p27^kip1^, and p57^kip2^ are all present in fetal germ cells in the testis at E14.5 (Western et al. 2008). In this strain, the RNA binding-protein dead end 1 (DND1) and inhibin α (INHA) are able to induce p21^cip1^ and p27^kip1^ in prospermatogonia to maintain dormancy (Western 2009; Cook et al. 2011; Mendis et al. 2011). In contrast, the investigators note that p21^cip1^ and p27^kip1^ proteins are absent in the prospermatogonia in the C57BL/6 strain at E14.5, which is corroborated by other studies (Beumer et al. 1999; Western et al. 2008). Interestingly, p27^kip1^ is localized to the prospermatogonia nucleus just prior to birth in C57BL/6 mice (Beumer et al. 1999). One explanation for this strain-specific difference in Cip/Kip expression may be that strain-specific inbred mutations trigger cell cycle checkpoints that change the timing of p21^cip1^ and p27^kip1^ expression in the germ cells. This delay, in C57BL/6 mice in this case, can be overcome and does not impact the progression of the prospermatogonia throughout development. This description is supported by data that show the role of p27^kip1^ in the nucleus is not crucial to cell cycle arrest, because prospermatogonia are still able to arrest at the appropriate stage in p27^kip1^-null mice (Beumer et al. 1999). The factors responsible for the activation of p27^kip1^ in the arrested prospermatogonia have yet to be determined. Making use of recent single-cell sequencing studies performed in the embryonic testis, both in the CD1 strain (Stévant et al. 2018, 2019) and in the C57BL/6 strain (Tan et al. 2020), may allow for the identification of genes that are expressed at the same time as p21^cip1^ and p27^kip1^ in the germ cells. In-depth analyses of these data sets together could yield interesting information of the signal that initiates Cip/Kip expression in the germ cells and where this signal originates from. Do intrinsic germ cell factors like DND1 or INHA induce prospermatogonia expression of p27^kip1^, or is this due to extrinsic factors from the Sertoli cell lineage activating pathways in the prospermatogonia nucleus (Nicol et al. 2018)? Manipulating genetic networks active in germ cells often proves challenging, because the mice become infertile. Alternative approaches, such as the conditional deletion of SOX9 at different time points in the Sertoli cells, might reveal whether the XY somatic cell environment is critical for the induction of p27^kip1^ in the gonocyte. However, this type of experiment may be difficult to interpret, either because functional redundancy between Sox9 and Sox8 may not lead to a sufficient change in the Sertoli cells, or (and particularly if both genes are mutated) a sudden change to a granulosa cell fate would be too disruptive. Alternatively, examination of p27^kip1^ nuclear expression in the XY testis with ectopic FOXL2 expression (Nicol et al. 2018) might indicate that the somatic cells are inducing gene changes in the gonocyte. Conversely, making use of conditional deletion tools to delete DND1 and INHA specifically in the gonocyte would uncover whether these factors, in combination with p27^kip1^, are critical for inducing key genetic changes that promote the production and development of a mature spermatozoon. This may give insight into novel genetic factors that disrupt the development of healthy spermatozoa and contribute to male infertility.

In the developing ovary, initial studies proposed that following sex determination, the decreased growth rate of XX gonads compared with XY gonads is due to the high expression of Cip/Kip cell cycle inhibitors (Nef et al. 2005). However, no embryonic ovarian defects are found in mouse models where p21^cip1^, p27^kip1^, or p57^kip2^ are genetically ablated (Nakayama and Nakayama 1998; Rajareddy et al. 2007). In addition, the ovaries in these three global deletion models do not match the fast growth rate or the size of the male testis, and moreover, p21^cip1^-null mice and p57^kip2^-null female mice are fertile (Nakayama and Nakayama 1998). These data support the concepts of redundancy between multiple dormancy factors, and/or the presence of a gene regulatory network with cofactors that regulate each other. These ideas are highly plausible in the context of the ovary, where several factors determine ovarian fate cooperatively, compared with the activation of single genes (*Sry* and then *Sox9*) in the testis (Fig. 4; Mork et al. 2012; Maatouk et al. 2013; Pannetier et al. 2016; Stévant and Nef 2019).

**Figure 6.**
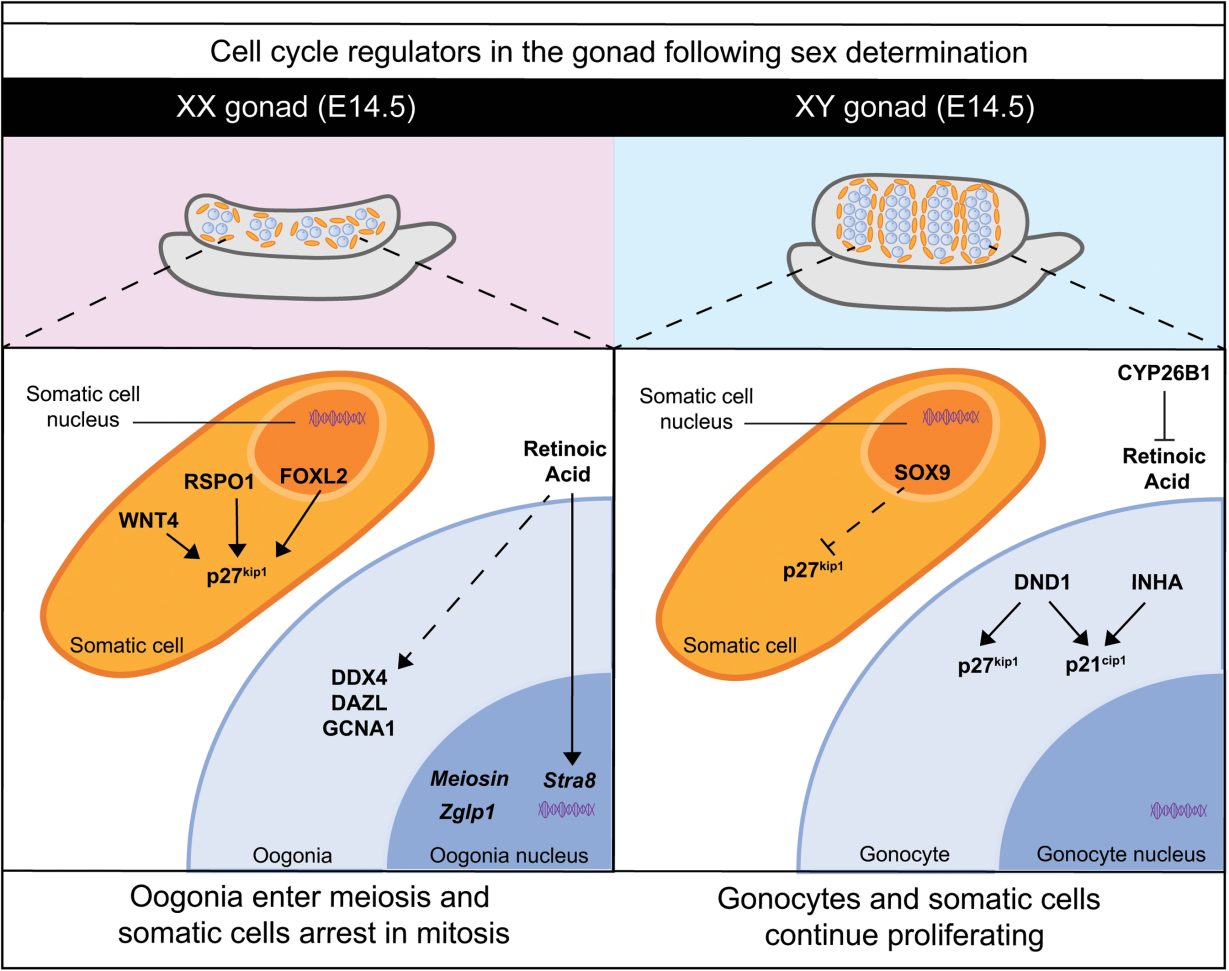
Differential control is exerted over cell cycle regulators in the gonad following sex determination. By E14.5 in XX gonads, the pregranulosa cells stop dividing. Factors involved in ovary development, such as FOXL2, WNT4, and RSPO1, have all been shown to activate p27kip1 to maintain the somatic cells in quiescence (Maatouk et al. 2013; Nicol et al. 2018; Richardson et al. 2020). In the XX gonad, oogonia enter and then arrest in meiosis, thereby establishing the ovarian reserve, although many oogonia are lost through atresia before birth. Retinoic acid, and perhaps other factors, stimulates the expression of Stra8 in the nucleus, which then results in the up-regulation of several critical meiotic regulators, including DDX4, DAZL, and GCNA1 (Bowles and Koopman 2010). Meiosin and Zglp1 are two factors recently shown to be important for activating the oogenic fate in the oogonia nucleus (Ishiguro et al. 2020; Nagaoka et al. 2020). In contrast, in the XY gonad there is continued proliferation of somatic cells (including Sertoli cells) and gonocytes, although the latter undergo cell cycle arrest (G_0_/G_1_) at about E13.5, as they become prospermatogonia, resuming mitotic cell divisions after birth (Jiang et al. 2019; Ruthig et al. 2019; Yan et al. 2020). With SOX9 expression in Sertoli cells, p27kip1 expression is repressed, allowing for cell cycle divisions (Nicol et al. 2018). CYP26B1, also made by Sertoli cells, inhibits the action of retinoic acid to prevent the gonocytes in the developing testis from entering meiosis (Bowles et al. 2006). Within the gonocyte, DND1 and INHA activate p27kip1 and p21cip1 to limit the proliferation of the germ cell pool to prevent the formation of teratomas (Mendis et al. 2011; Cook et al. 2011; Ruthig et al. 2019).

Within the embryonic ovary, p27^kip1^ is restricted initially to the somatic cells that become the granulosa cells postnatally, and p27^kip1^ expression is only found in the oocytes around postnatal day (PND) 4 (Rajareddy et al. 2007). The expression of p27^kip1^ in somatic cells is maintained by the female-determining factors, including WNT4, RSPO1, and FOXL2, in the E14.5 mouse ovary (Fig. 6; Fero et al. 1996; Maatouk et al. 2013; Gustin et al. 2016; Richardson et al. 2020). Interestingly, in WNT4-null mice, p27^kip1^ is specifically down-regulated at the anterior pole of the ovary at E15.5 (Maatouk et al. 2013). This area of p27^kip1^ down-regulation shows an up-regulation of the male factor anti-Müllerian hormone (AMH), which is testis-specific before birth but is expressed by granulosa cells in the postnatal ovary (Maatouk et al. 2013). In the days following sex determination, up-regulation of p27^kip1^ expression in the pregranulosa cells coincides with the entry of oogonia into meiosis, thus establishing the germ cell pool. Conditional deletion of p27^kip1^ from the pregranulosa cell lineage would confirm whether an interaction exists between p27^kip1^ in the pregranulosa cells and the initiation of meiosis in the germ cells, thus linking pregranulosa cell signaling to cell cycle control in the female germ cells.

## Oogonia enter meiosis shortly after sex determination

The establishment of the germ cell pool during ovarian development in mammals is necessary to ensure continued fertility in adulthood (Fig. 6). Although there can be germline stem cells in lower vertebrates, all of the oocytes that a female mammal will ever ovulate originate from the reservoir of oogonia established by the end of gestation (Sarraj and Drummond 2012). These are all arrested in meiosis I, and at present there is limited evidence, none of which is compelling, to suggest that new oocytes are able to be generated postnatally in vivo (McGee and Hsueh 2000; Woods and Tilly 2013; Navaroli et al. 2016; Zarate-Garcia et al. 2016; Wagner et al. 2020). This ultimately means that the female germ cell pool must rapidly proliferate during gestation, such that by the end of gestation there remains a sufficient number of oocytes (∼8000 oocytes in mice) (Kerr et al. 2006) to establish and maintain this reserve for the entirety of the female reproductive life span (McGee and Hsueh 2000; Skinner 2005; Adhikari and Liu 2009; McLaughlin and McIver 2009). Given that the female germ cell pool must proliferate rapidly over a short gestational time, it is assumed that the cyclin-dependent kinase inhibitors are down-regulated. Indeed, p27^kip1^ is not detected by immunofluorescence in the female germ cells between E12.5 and E14.5 in the developing ovaries (Rajareddy et al. 2007; Mork et al. 2012). It would be valuable to determine whether down-regulation of cyclin-dependent kinase inhibitors in the oogonia is necessary to facilitate the switch to meiosis. Following E12.5, after sex determination, there are two major differences between germ cells in the ovary and in the testis. In the male, germ cell cysts must break down in a manner to retain a high proportion of germ cells, which go on to become germline stem cells (Lei and Spradling 2013b). This requires the male germ cells to arrest in mitosis, which may be facilitated by cell cycle inhibitors. The second major difference between germ cells in the ovary and in the testis is the decision to enter meiosis early or to arrest in mitosis (Bowles and Koopman 2010).

Germ cell sex is not controlled by the presence of XX or XY chromosomes within the germ cells; instead, they rely on the surrounding somatic environment for their commitment to oogenesis or spermatogenesis (Adams and McLaren 2002). Watershed coculture studies, where E11.5 LacZ-labeled XX germ cells were cultured with E12.5 XY genital ridges, showed that XX germ cells would, rather than switch to meiosis, continue in mitosis in the XY genital ridge (Adams and McLaren 2002). There are also studies in chimeras and in sex-reversed mice, both XX male and XY female, that germ cell sex is dependent on the type of gonad, i.e., ovaries or testes, rather than on their own sex chromosome status (Ottolenghi et al. 2007; Vernet et al. 2014). These results showed that signals from the surrounding somatic environment stimulate and drive the switch from mitosis to meiosis.

Multiple signals emanate from the somatic environment to promote meiosis. The classical pathway is the retinoic acid pathway, where retinoic acid (RA) released from the mesonephros into the gonad is responsible for stimulating meiosis within the ovary (Bowles et al. 2006). Expression of the premeiotic marker *Stra8* and up-regulation of the meiotic markers *Sycp3* and *Dmc1* are stimulated by RA signaling (Fig. 6; Bowles et al. 2006). New studies suggest that it may not be retinoic acid, with meiosis occurring normally in mice without any retinoic acid receptors (Vernet et al. 2020). Additionally, new pathways responsible for stimulating the oogonia to enter meiosis are emerging. For example, a recent study identified MEIOSIN as a cofactor of STRA8, where MEIOSIN and STRA8 bind to the transcriptional start sites of target genes, allowing for the rapid transcription that is required of germ cells entering meiosis (Ishiguro et al. 2020). Another factor, ZGLP1, precedes the expression of *Stra8*, and Zglp1^−/−^ mice have atrophied ovaries with no oocytes as early as E17.5 (Nagaoka et al. 2020). Promising data show that ZGLP1 is downstream from BMP2 in primordial germ cell-like cells (PGCLCs), linking the in vitro model to the in vivo mouse model (Nagaoka et al. 2020). These data follow on from recent work that described a mechanism for meiosis entry where BMP is also required alongside RA for germ cell sex specification (Miyauchi et al. 2017). Both MEIOSIN and ZGLP1 are essential for stimulating the oogenic program, but it is unclear what signals are activating these factors in vivo. Further work to examine how BMP and RA coordinate meiosis entry will provide more detailed networks that promote the activation of meiosis at specific temporal windows.

A major challenge in studying the mitosis-to-meiosis switch in mice is that their germ cells enter meiosis asynchronously, in an anterior-to-posterior pattern (Bullejos and Koopman 2004). To enter meiosis and become a functional gamete, germ cells must also turn off pluripotency factors, including NANOG and OCT4 (Feng et al. 2014; Jørgensen and Rajpert-De Meyts 2014; Arora et al. 2016). Over a 4-d period, between E12.5 and E16.5, the oogonia switch off OCT4 and up-regulate SYCP3 in an anterior-to-posterior wave (Niu and Spradling 2020). These subtle transcriptional changes cannot be detected using traditional global RNA sequencing methods. However, recent single-cell sequencing approaches have been successfully used to uncover other discreet spatial and temporal regulators of meiosis (Niu and Spradling 2020; Zhao et al. 2020). Both studies performed fine-scale analyses of the germ cell meiotic transcriptomes covering the E12.5 and E14.5 (Niu and Spradling 2020) and E12.5, E14.5, and E16.5 (Zhao et al. 2020) time points, respectively. Another unreviewed preprint also analyzed germ cell transcriptomes between E10.5 and E16.5 (Mayère et al. 2021). All the studies conclude that XX and XY germ cells diverge around E12.5 and that thousands of genes varied during this period of development. By analyzing these extensive data sets, multiple pathways and gene regulatory networks will be identified that are important for initiating and progressing meiosis.

Together with the information obtained about the initiation of meiosis, these data sets have also provided a wealth of information about Cip/Kip family expression between E12.5 and E16.5 in mice (Niu and Spradling 2020; Zhao et al. 2020). Both studies showed that *Cdkn1a* expression peaks in the preleptotene stage of germ cell development and decreases as the germ cells enter the leptotene stage of meiosis (Niu and Spradling 2020; Zhao et al. 2020). In contrast, *Cdkn1c* expression peaks at the leptotene stage, before decreasing as the germ cells continue through meiosis (Niu and Spradling 2020). Interestingly, *Cdkn1b* transcript is expressed stably throughout the stages of meiosis, despite not being detected by immunofluorescence (Rajareddy et al. 2007; Niu and Spradling 2020). Alternatively, both *Cdkn1a* and *Cdkn1c* decrease as the germ cells enter meiosis; however, it is unknown whether this is a contributing factor to meiosis entry or is a consequence of the change in the cell division process.

## Communication between the oogonia and somatic cells establishes germ cell nests in the ovary

**Figure 7.**
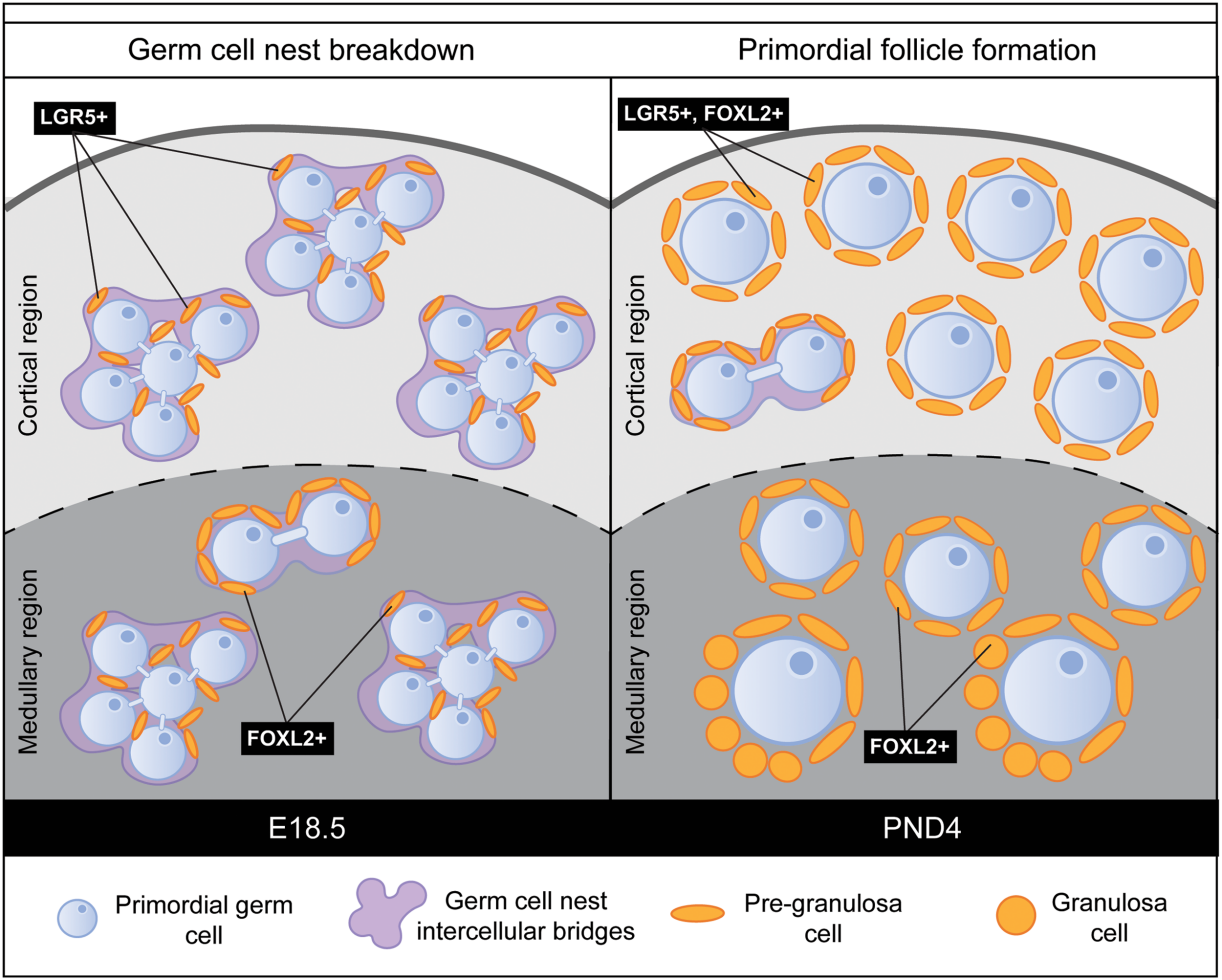
Germ cell nest breakdown and primordial follicle formation are asynchronous events. During embryonic development, XX primordial germ cells arrest in the diplotene stage of prophase and are termed oogonia. By E18.5, these oogonia are now termed oocytes and are surrounded by intercellular bridges (purple) and somatic pregranulosa cells (orange). This structure is termed a germ cell nest. The process of germ cell nest breakdown is asynchronous, with medullary nests broken down first. Germ cell nests begin to be broken down before birth, and the somatic pregranulosa cells (orange) infiltrate the nest and surround the oocyte, through a process known as primordial follicle formation. A primordial follicle is an oocyte surrounded by one layer of flattened pregranulosa cells. Primordial follicles remain dormant until the process of primordial follicle activation, where follicles are selected to develop. This process also begins first in the medullary region of the ovary around PND4, and cortical follicles remain quiescent.

As the ovary continues to develop, the oogonia rely primarily on the ovarian environment, established by the pregranulosa cells. The somatic pregranulosa cells contribute to the structure, known as a germ cell nest, which contains the oogonia (Fig. 7; Pepling and Spradling 1998, 2001). Nest formation is a lengthened process, where cysts begin to form around E10.5 and E11.5, and finish assembling around E13.5–E14.5, through five rounds of divisions of the oogonia (Lei and Spradling 2016). Much like the onset of meiosis, germ cell nest formation is asynchronous (Lei and Spradling 2016; Wang et al. 2017). Due to this asynchronous nature of germ cell nest formation, little is known about the pathways responsible that guide the formation of the nests. However, communication between JAGGED1 in the oocytes and NOTCH2 in the pregranulosa cells is considered important in regulating nest formation (Xu and Gridley 2013; Vanorny et al. 2014). Each nest holds several somatic cells, as well as germ cells connected by intercellular bridges, which join the cytoplasm of individual germ cells within a cyst (Lei and Spradling 2013b, 2016). In the germ cells at least, canonical cell cycle machinery must be silenced to block cytokinesis and allow intercellular bridge formation (Lei and Spradling 2013b). How the germ cells are programmed to cease cycling and generate germ cell cyst structures warrants further study but may be due to the broad action of STRA8 (Kojima et al. 2019). Large cysts separate into smaller cysts such that, by E17.5, cysts contain between three and four oogonia, and at birth cysts only contain two oogonia (Lei and Spradling 2013a). It is thought that cytokinesis remains incomplete to form the intercellular bridges within each cyst (Lei and Spradling 2013b). However, the direct mechanisms that drive the breakdown of these cysts are not known in detail. Pioneering studies from Lei and Spradling (2016) have contributed the most to this question, showing that cyst breakdown is dependent on a number of events, including the fusion of germ cell membranes, the transfer of cytoplasmic materials between germ cells, and the apoptosis of remnant materials and nuclei. As the mechanisms governing germ cell nest breakdown are still being elucidated, little is known about the expression of the Cip/Kip family during germ cell nest formation and breakdown. One observation in p27^kip1^-null mice is an accelerated assembly of primordial follicles, where at PND1, oogonia are already separated from the nest structure and pregranulosa cells are surrounding the oogonia, much earlier than in ovaries from wild-type littermates (Rajareddy et al. 2007). These mice also have higher rates of multi-oocyte follicles, indicating a defect in the formation of primordial follicles (Perez-Sanz et al. 2013). However, this phenotype is observed in many gene deletion studies and through the use of microtubule inhibitors, both in vivo and in vitro, and therefore it may not be specific to cell cycle interferences. This highlights that the mechanisms in germ cell nest formation and breakdown that drive these ovarian phenotypes are unknown and require further examination.

## Germ cell nest breakdown leads to the formation of primordial follicles

A large body of work has contributed to a detailed understanding of the process of germ cell nest breakdown (for reviews on this topic, see Tingen et al. 2009; Wear et al. 2016; Ikami et al. 2017). This process underpins another key stage of ovarian development, as nest breakdown is the first step in the formation of primordial follicles (Tingen et al. 2009; Suzuki et al. 2015; Rosario et al. 2019). Primordial follicles are the structures that maintain oocytes in dormancy after birth and into adulthood (Skinner 2005). A primordial follicle is composed of an oocyte surrounded by one layer of flattened pregranulosa cells (Fig. 7; McLaughlin and McIver 2009; Tingen et al. 2009; Reddy et al. 2010; Sánchez and Smitz 2012; Sarraj and Drummond 2012). Proper formation of the primordial follicles relies on the specific temporal initiation of germ cell nest breakdown (Hardy et al. 2018; Ford et al. 2019; Chakravarthi et al. 2020). Germ cell nest breakdown is initiated around E17.5 in the medullary region of the ovary. Nest breakdown then continues over a period of ∼5 d, with medullary cysts the first to form primordial follicles. Cortical cysts break down after birth, ∼3 d postpartum (Wang et al. 2017). Over this time, pregranulosa cells invade the germ cell nests, and the intercellular bridges between germ cells are broken down (Pepling and Spradling 2001; Lei and Spradling 2013b).

Many of the canonical pathways in intercellular bridge breakdown are common to those involved in follicle formation, including NOTCH signaling, E-cadherin, KITL, and TGF-β signaling (Chen et al. 2007; Jones and Pepling 2013; Xu and Gridley 2013; Wang et al. 2015). These pathways have ligands and receptors in both granulosa cells and oocytes that signal to each other to coordinate germ cell nest breakdown. Recently, novel transcription factors have been found to stimulate and facilitate germ cell nest breakdown. SP1, IRX3, and IRX5 are all factors expressed in pregranulosa cells that promote germ cell nest breakdown (Fu et al. 2018; Cai et al. 2019). When SP1 is lost, germ cell nests persist and primordial follicle formation does not occur, leading to an infertility phenotype after birth (Cai et al. 2019). Irx3 is specifically expressed in the intercellular bridges of germ cell nests, and when both Irx3 and Irx5 are deleted, defective granulosa cells fail to connect with the oocyte, causing aberrant follicle formation and apoptosis of the developing follicles (Fu et al. 2018). Other factors are specific to the signals originating from the oocyte, including factors like ELAV-like protein 2 (ELAVL2) and DEAD-box helicase 6 (DDX6) (Kato et al. 2019). Loss of ELAVL2 or DDX6 in isolation both lead to an infertility phenotype in mice, with no oocytes remaining in the ovary 2 wk after birth due to a defect in primordial follicle formation (Kato et al. 2019). Together these studies show that the maintenance of granulosa–oocyte communication is essential for germ cell nest breakdown and follicle formation. Importantly, if this communication is disrupted or fails, there are major consequences for future fertility.

## Single-cell sequencing data sets provide new insights into germ cell nest breakdown and primordial follicle formation

Due to the intricate communication networks that exist between oocytes and the granulosa cells, it has proven difficult to isolate the group of cells responsible for the initiation of germ cell nest breakdown and follicle formation. Pioneering single-cell sequencing experiments provide alternative methods to lineage-tracing studies. Using single-cell sequencing, individual cells are clustered together into related populations based on transcriptomic data. In the ovary, the pregranulosa cell lineages and subclusters have been reconstructed using single-cell data sets (Niu and Spradling 2020; Wang et al. 2020). Comprehensive data sets covering the stages of germ cell nest breakdown, primordial follicle formation, and primordial follicle activation were generated using single-cell sequencing on whole ovaries from E18.5, PND1, and PND5 (Niu and Spradling 2020). Unlike the single-cell sequencing data sets from Stévant et al. (2018, 2019), which isolated cells using the expression of SF1, this study allows the simultaneous comparison of germ cells and somatic cells. Preliminary findings from this study reveal key insights into ovarian development. Namely, that germ cells are uniform early in development and validate the independent origins of several distinct populations of pregranulosa cells (Niu and Spradling 2020). One of the key contributions of this work to our knowledge is that two distinct classes of primordial follicles are formed during germ cell nest breakdown and that these primordial follicles contain pregranulosa cells with different gene expression signatures (Niu and Spradling 2020). These two classes of primordial follicles also vary in their cell cycle status, where medullary wave 1 follicles immediately activate and the granulosa cells begin proliferating, whereas cortical wave 2 follicles remain arrested (Niu and Spradling 2020). It would be interesting to examine the regulatory mechanisms controlling the resumption of the cell cycle in wave 1 follicles. Importantly, in the Niu and Spradling (2020) data set, all three Cip/Kip family members *Cdkn1a*, *Cdkn1b*, and *Cdkn1c* are all expressed in both pregranulosa populations, whether the cells are of a bipotential cell origin or an epithelial cell origin. A subsequent study from Wang et al. (2020) performed a similar study at E16.5, PND0, and PND3 to better understand the dynamics of primordial follicle assembly. This study confirmed the presence of multiple populations of pregranulosa cells and went one step further to examine the transcriptional networks that are involved in the interactions between oocytes and pregranulosa cells (Wang et al. 2020). In agreement with the Niu and Spradling (2020) study, Wang et al. (2020) comment on the expression of *Cdkn1a* and *Cdkn1b* in the pregranulosa cells and hypothesize an interaction with the FOXO pathways in oocytes (Wang et al. 2020).

The identification of multiple populations of pregranulosa cells has been reported before, although single-cell sequencing allows the interrogation of these cell types in great detail at the transcriptional level. Over the past 10 yr, studies have shown that two classes of primordial follicles are formed during early postnatal life. Mounting evidence shows heterogeneity within the pregranulosa cell population, indicating that the SF1-positive lineage from the bipotential gonad is not the only source of pregranulosa cells (Mork et al. 2012; Rastetter et al. 2014; Nicol et al. 2018; Stévant et al. 2019). For example, cells from the ovarian surface epithelium migrate into the ovarian cortex and differentiate into pregranulosa cells (Mork et al. 2012). The epithelial-derived pregranulosa cells remain near the epithelium in the cortex of the ovary and express leucine-rich repeat-containing G protein-coupled receptor 5 (LGR5) (Ng et al. 2014; Rastetter et al. 2014). These two populations of pregranulosa cells remain restricted to distinct domains of the ovary to contribute to two classes of primordial follicles. The inner ovarian medullary region contains germ cells surrounded by pregranulosa cells positive for FOXL2 expression, and the outer ovarian cortical region contains migrating pregranulosa cells from the epithelium positive for LGR5 expression, and later express FOXL2 (Rastetter et al. 2014). The distinction between these populations of primordial follicles is very important, as medullary primordial follicles activate for development just after birth (Mork et al. 2012; Maatouk et al. 2013). In contrast, cortical primordial follicles maintain dormancy in the ovary and provide the oocytes for the reproductive life span of mice (Mork et al. 2012). To maintain fertility, these follicles must remain dormant, until temporally selected for the process of primordial follicle activation.

## Numerous pathways lead to a wave of primordial follicle activation postnatally

Primordial follicle activation is the process by which dormant follicles are selectively chosen for growth and development in preparation for ovulation. This overarching developmental process is known as folliculogenesis, and a number of current and comprehensive reviews detail this process (Kerr et al. 2013; Hsueh et al. 2015; Kallen et al. 2018; Ford et al. 2019). While new sperm are continuously produced during the reproductive life span of males, there is no generation of new oocytes in the ovary, and the primordial follicles that are assembled in the perinatal period are responsible for the entire reproductive life span of the adult female (McLaughlin and McIver 2009). Consequently, tight regulation of follicle activation is imperative to ensuring fertility. Activation of all primordial follicles at once or an inability to coordinate temporal follicle activation results in premature infertility. Numerous studies in mice have shown that, similar to germ cell nest breakdown, cellular cross-talk between oocytes and pregranulosa cells is essential for coordinating primordial follicle activation (Adhikari and Liu 2009). Well-characterized signaling pathways in the activation of primordial follicles include the phosphoinositide 3-kinase (PI3K) pathway, phosphatase and tensin homolog (PTEN) pathway, kit ligand (KIT/L) pathway, forkhead box L2 (FOXL2) pathway, and JAK/STAT pathway (Uda et al. 2004; Hutt et al. 2006; Reddy et al. 2008; Jagarlamudi et al. 2009; Sutherland et al. 2012, 2018; Hall et al. 2017; Grosbois and Demeestere 2018). These studies illustrate that it is unlikely for a singular pathway to stimulate or repress the activation of primordial follicles. Moreover, it is the coordinated interplay of numerous signaling pathways that regulates this pivotal developmental process.

## Cell cycle inhibitors maintain primordial follicles in quiescence

Factors that inhibit primordial follicle activation also play a critical role in maintaining fertility, and cell cycle inhibitors maintain pregranulosa cell identity in the dormant state by preventing cellular proliferation. Both p21^cip1^ and p27^kip1^ have been implicated in germ cell nest breakdown and primordial follicle formation (Fig. 7; Bayrak and Oktay 2003; Rajareddy et al. 2007). p21^cip1^ is regulated by the proliferation pathway member JAK2, which is known to facilitate germ cell nest breakdown (Huang et al. 2018). However, numerous studies show a function of p27^kip1^ in germ cell nest breakdown and follicle development (Fero et al. 1996; Bayrak and Oktay 2003; Rajareddy et al. 2007; Hirashima et al. 2011; Perez-Sanz et al. 2013). Global deletion of p27^kip1^ in mice causes several deficient ovarian phenotypes. First, accelerated activation of all primordial follicles occurs immediately after birth (Rajareddy et al. 2007). As no primordial follicles remain in the ovary by 12 wk of age, the mouse is infertile (Rajareddy et al. 2007). Second, as the ovary contains a larger proportion of multi-oocyte follicles, germ cell nest breakdown is perturbed (Perez-Sanz et al. 2013). This is attributed to a defect in pregranulosa cell migration into the cyst necessary to encircle and separate individual oocytes (Perez-Sanz et al. 2013). siRNA knockdown of p27^kip1^ in 3-d mouse ovaries also presents with a precocious activation of all the primordial follicles (Hirashima et al. 2011). Combined, these experiments describe an essential role for cell cycle inhibitors in postnatal ovary development. It is important to note that these studies are performed in mice with complete Cip/Kip deletions that are present from birth, rather than targeted conditional cell type-specific deletions or inducible deletions. As a result, the initiation of the defect is still unclear; for example, it is conceivable that the adult ovarian fertility phenotype persists from a germ cell defect at the time of cyst breakdown. Precise genetic experiments, such as a Cip/Kip-inducible deletion mouse line or a Cip/Kip deleted only in the germ cells (using a germ cell Cre line), would add valuable information to the field about the function of Cip/Kip during follicle formation and development. Using the single-cell sequencing techniques in mouse models where follicle activation is dysregulated, as in the p27-null mouse model, will also highlight the changes at the gene level and give an insight into how p27^kip1^ might control primordial follicle activation. Another question worth addressing is whether p27^kip1^ can also act as a transcription factor during follicle development to stimulate other dormancy factors to sustain oocytes for future fertility. The role of Cip/Kip members in follicle activation may be also an indirect effect of the follicle activation process. Loss of p27^kip1^ leaves several cyclins and cyclin-dependent kinases free to promote cell cycle progression, thus encouraging granulosa cell proliferation. Cyclin D is known to be directly regulated by SMAD3 (Granados-Aparici et al. 2019), which is a well-known inhibitor of follicle activation, implicating other cell cycle genes in primordial follicle activation. Further functional studies into the networks with which the cyclin-dependent kinase inhibitors interact will elucidate whether these factors are involved in novel pathways or in combination with well-established follicle activation pathways.

## Conclusions

Until recently, our knowledge about ovarian development has largely been limited to information derived from analyzing the expression and function of single genes. The advent of new sequencing technologies like single-cell RNA sequencing has revealed that complex networks stimulate early ovarian development and support the formation of primordial follicles. These networks work in cooperation with cell cycle regulators to ensure that proliferation and differentiation occur at the appropriate temporal window. These cell cycle regulators, including the Cip/Kip family, work in opposing ways in the germ cell and supporting cell lineages. In the male, proliferation of the germ cells and supporting cell lineages is stimulated to produce a testis capable of generating large numbers of spermatozoa. In the male, Cip/Kip expression is decreased after sex determination to allow for this rapid growth of the testis. In the female, however, Cip/Kip expression increases to restrict germ cell proliferation and ensure that there are enough supporting cells to sustain oocyte maturation in the adult ovary. To date, single-cell sequencing technologies have been applied in embryonic ovaries. Application of these sequencing technologies across a wider range of time points in ovarian development has the potential to yield huge insights in oocyte maturation and ovarian aging. An enhanced understanding of follicle formation and activation in mammals will form the basis for the development of novel fertility preservation techniques and the diagnosis of ovarian infertility.
